# Supplementary material for: Local weakening of cell-extracellular matrix adhesion triggers basal epithelial tissue folding
Source: EMBO J. 2025 Feb 17;44(7):2002–24. doi: 10.1038/s44318-025-00384-6 (PMC11961693; doi:10.1038/s44318-025-00384-6)
Supplement: Supplementary file 1 — Appendix [file 44318_2025_384_MOESM1_ESM.pdf]

Table of contents

Table of contents	Page
Appendix Figure S1 A wild type third instar wing disc.	2
Appendix Figure S2 P-Myosin distribution in the wing margin changes over Development.	3
Appendix Figure S3 Downregulation of myosin activity prevents basal tissue indentation.	4
Appendix Figure S4 Integrins overexpression in the wing margin does not affect cell fate.	5

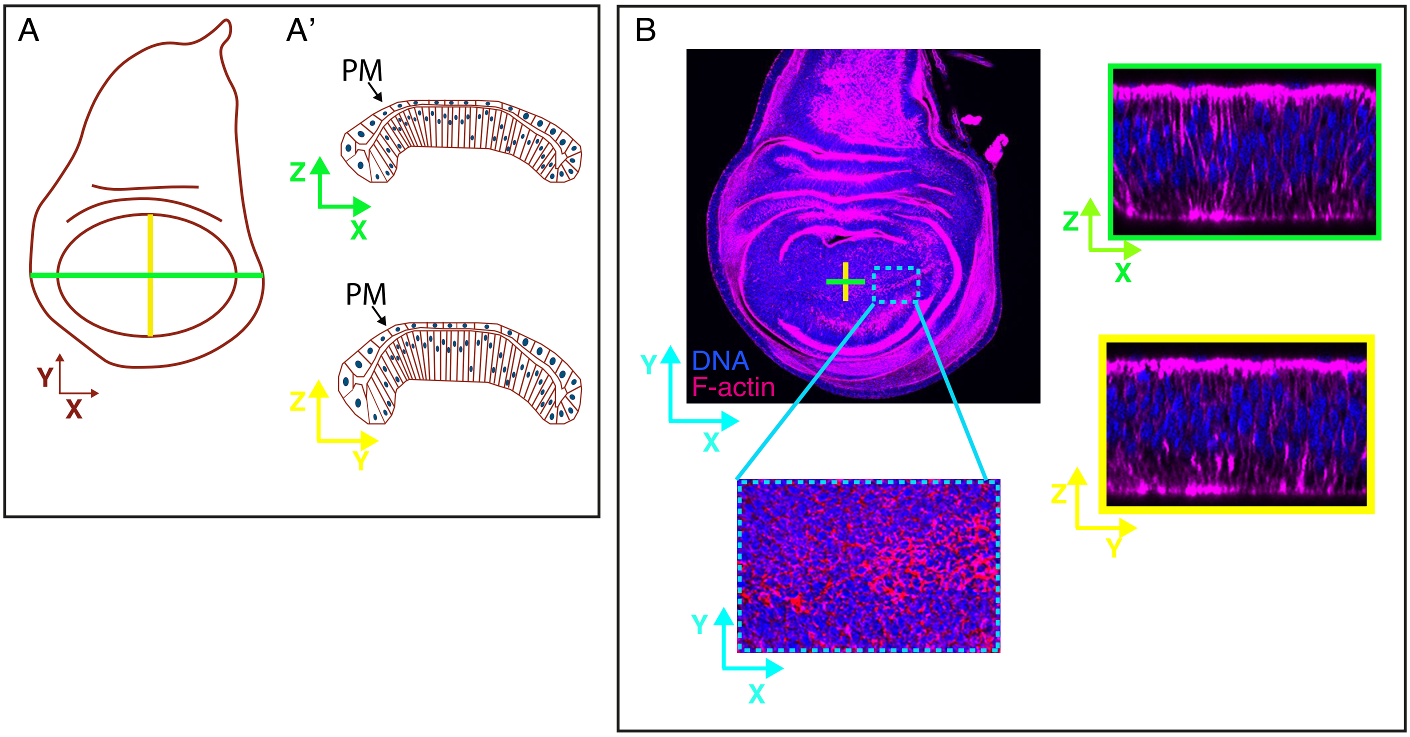

Appendix Figure S1 A wild type third instar wing disc.
(A) Scheme of a wild type third instar wing disc and (A’) ZX (upper) and ZY (lower) cross sections along green and yellow lines in A.
(B) Confocal view of third instar wing imaginal discs stained with the F-actin marker Rhodamine Phalloidin (magenta) and the nuclear marker Hoechst (DNA, blue) and cross-sections along the indicated axis.

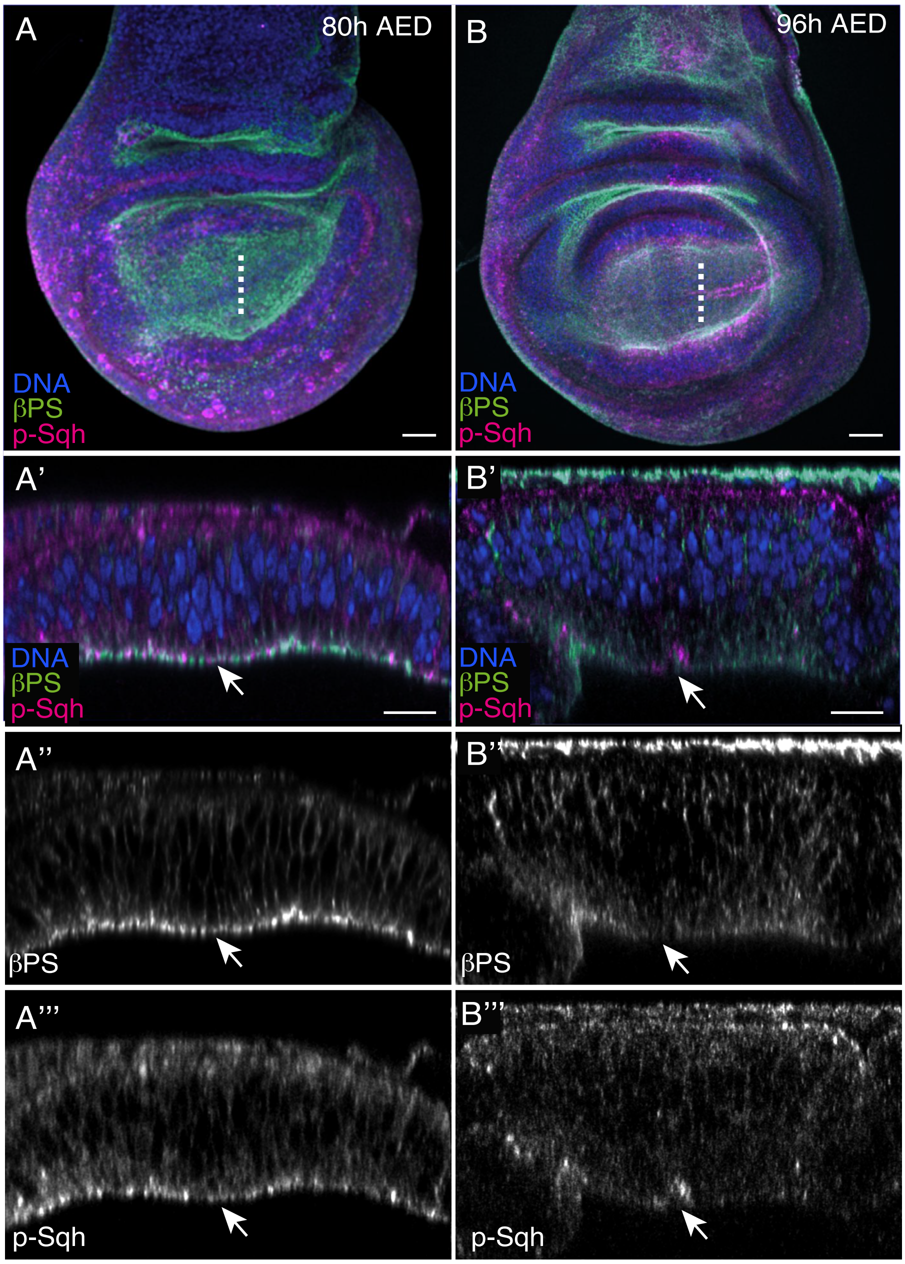

Appendix Figure S2 P-Myosin distribution in the wing margin changes over development.

(A-B’’’) Confocal views of third-instar wing discs at 80h AED (A) and 96h AED (B) stained with anti-βPS (green in A-B’ and white in A’’, B’’), anti-pSqh (magenta in A-B’ and white in A’’’, B’’’) and the nuclear marker Hoechst (DNA, blue in A–B’). (A’-B’’’) Confocal YZ cross-sections along the white dotted lines shown in (A, B). White arrows in (A’- B’’’) point to the wing margin region. Scale bar in all panels, 30µm. At least 15 wing discs were assessed over three independent experiments.

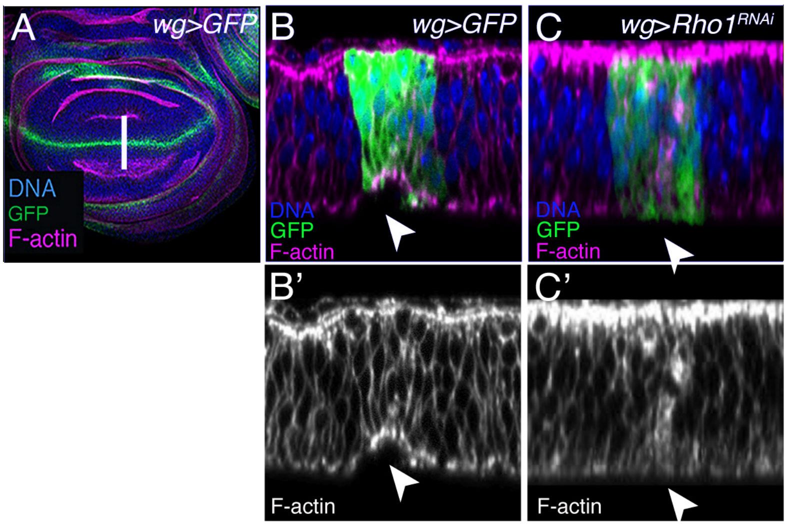

Appendix Figure S3. Downregulation of myosin activity prevents basal tissue indentation.

(A) Confocal view of a 3rd instar wing disc expressing GFP under the wgGal4 line (A, wg>GFP), stained with anti-GFP (green), the F-actin marker Rhodamine-Phalloidin (magenta) and the nuclear marker Hoechst (blue). (B-C') Confocal YZ cross-section along the white line in A of wing discs of the designated genotypes stained with anti-GFP (green in B, C), Rhodamine-Phalloidin (magenta in B, C, white in B', C') and Hoechst (blue in B, C). White arrows in B-C' point to the wing margin fold.

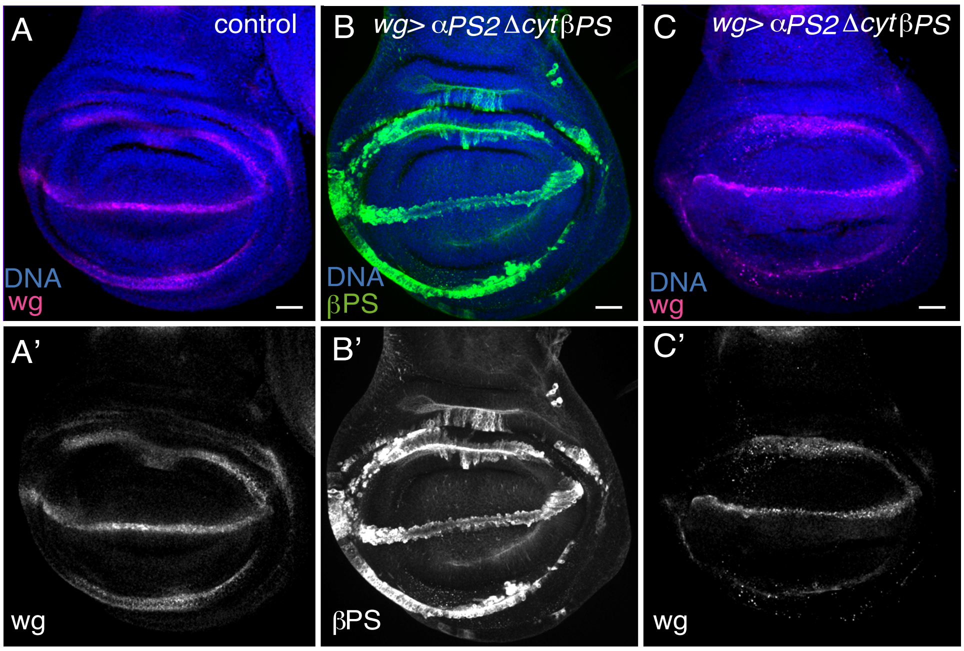

Appendix Figure S4. Integrins overexpression in the wing margin does not affect cell fate.

(A-C’) Maximal projections of confocal views of wing imaginal discs of third-instar larvae stained with anti-wg (magenta in A, C and white in A’, C’), anti-βPS (green in B and white in B’) and the nuclear marker Hoechst (DNA, blue). (A) Control wing disc. (B, C) Wing disc co-expressing an active form of the αPS2 subunit and the βPS subunit under the control of wgGal4, wg>αPS2ΔCyt; βPS. Scale bar in all panels, 30µm.
