# Supplementary material for: Local weakening of cell-extracellular matrix adhesion triggers basal epithelial tissue folding
Source: EMBO J. 2025 Feb 17;44(7):2002–24. doi: 10.1038/s44318-025-00384-6 (PMC11961693; doi:10.1038/s44318-025-00384-6)
Supplement: Supplementary file 3 — Movie EV1 [file 44318_2025_384_MOESM3_ESM.zip › Legend Movie EV1.docx]

**Movie EV1.** **Combined action of reducing integrin adhesion strength and increasing basolateral contraction triggers basal folding.**

Simulation of a simultaneous reduction in integrin adhesion strength and increase in basolateral contractility (related to Fig.3C). Integrin adhesion weakening in the wing margin was modelled as a change in stiffness from 160 kPa to 0.000001 kPa. The increase in basolateral contractility in the wing margin region was modelled as a 40% decrease in cell height. Simulation time is shown on the top left corner of the movie.
