# Supplementary material for: Local weakening of cell-extracellular matrix adhesion triggers basal epithelial tissue folding
Source: EMBO J. 2025 Feb 17;44(7):2002–24. doi: 10.1038/s44318-025-00384-6 (PMC11961693; doi:10.1038/s44318-025-00384-6)
Supplement: Supplementary file 4 — Movie EV2 [file 44318_2025_384_MOESM4_ESM.zip › Legend Movie EV2.docx]

**Movie EV2 Integrin adhesion weakening must precede basolateral contraction to allow proper basal folding initiation.**

Simulation of decreasing integrin adhesion strength prior to increasing basolateral contractility (related to Fig.4A). Integrin adhesion weakening, modelled as a change in stiffness from 160 kPa to 0.000001 kPa, was applied from 0 to 8h. The increase in basolateral contractility, simulated as a 40% decrease in cell height, was applied from 8 to 40h. Simulation time is shown on the top left corner of the movie.
