# Supplementary material for: Local weakening of cell-extracellular matrix adhesion triggers basal epithelial tissue folding
Source: EMBO J. 2025 Feb 17;44(7):2002–24. doi: 10.1038/s44318-025-00384-6 (PMC11961693; doi:10.1038/s44318-025-00384-6)
Supplement: Supplementary file 5 — Movie EV3 [file 44318_2025_384_MOESM5_ESM.zip › Legend Movie EV3.docx]

**Movie EV3 Basolateral contractility is necessary to initiate basal folding**.

Simulation of just reducing integrin adhesion strength without changing basolateral contractility (related to Fig.6F). Integrin adhesion weakening in the wing margin was modelled as a change in stiffness from 160 kPa to 0.000001 kPa. Simulation time is shown on the top left corner of the movie.
