# Supplementary material for: Local weakening of cell-extracellular matrix adhesion triggers basal epithelial tissue folding
Source: EMBO J. 2025 Feb 17;44(7):2002–24. doi: 10.1038/s44318-025-00384-6 (PMC11961693; doi:10.1038/s44318-025-00384-6)
Supplement: Supplementary file 6 — Movie EV4 [file 44318_2025_384_MOESM6_ESM.zip › Legend Movie EV4.docx]

**Movie EV4 Preventing integrin adhesion weakening and basolateral contractility hinders initiation of basal folding.**

Simulation of a situation where no change was applied to either integrin adhesion strength or basolateral contractility (related Fig.7F). Stiffness was 160 kPa. Simulation time is shown on the top left corner of the movie.
