# Supplementary material for: Local weakening of cell-extracellular matrix adhesion triggers basal epithelial tissue folding
Source: EMBO J. 2025 Feb 17;44(7):2002–24. doi: 10.1038/s44318-025-00384-6 (PMC11961693; doi:10.1038/s44318-025-00384-6)
Supplement: Supplementary file 7 — Movie EV5 [file 44318_2025_384_MOESM7_ESM.zip › Legend Movie EV5.docx]

**Movie EV5 Integrin adhesion weakening is necessary to trigger initiation of proper folding in the wing margin.**

Simulation of just increasing basolateral contractility without changing integrin adhesion strength (related to Fig.7G). Stiffness was 160 kPa. The increase in basolateral contractility in the wing margin region was modelled as a 40% decrease in cell height. Simulation time is shown on the top left corner of the movie.
