# Supplementary figures and images for: Local weakening of cell-extracellular matrix adhesion triggers basal epithelial tissue folding
Source: EMBO J. 2025 Feb 17;44(7):2002–24. doi: 10.1038/s44318-025-00384-6 (PMC11961693; doi:10.1038/s44318-025-00384-6)

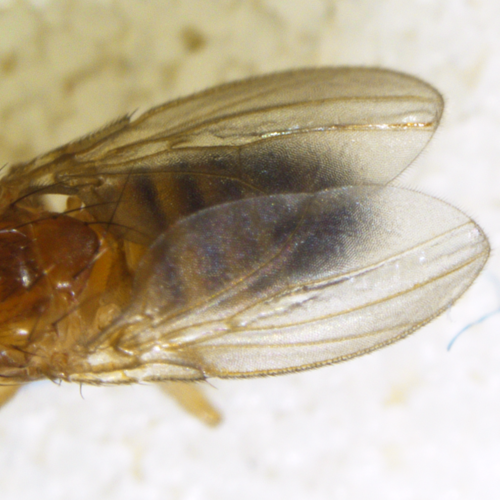

Supplement: Supplementary file 15 — Source data Fig. 8 [file 44318_2025_384_MOESM15_ESM.zip › Figure 8/fig8_j.tif]

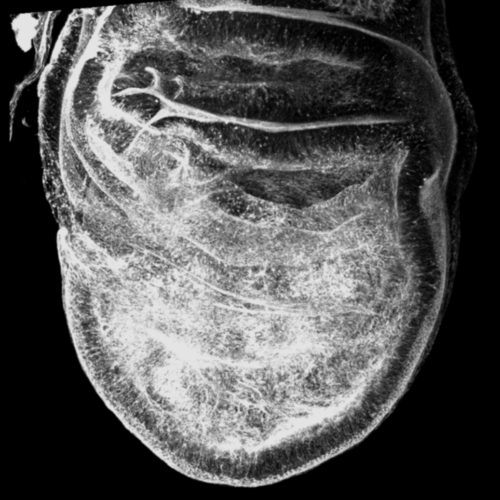

Supplement: Supplementary file 15 — Source data Fig. 8 [file 44318_2025_384_MOESM15_ESM.zip › Figure 8/fig8_i.tif]

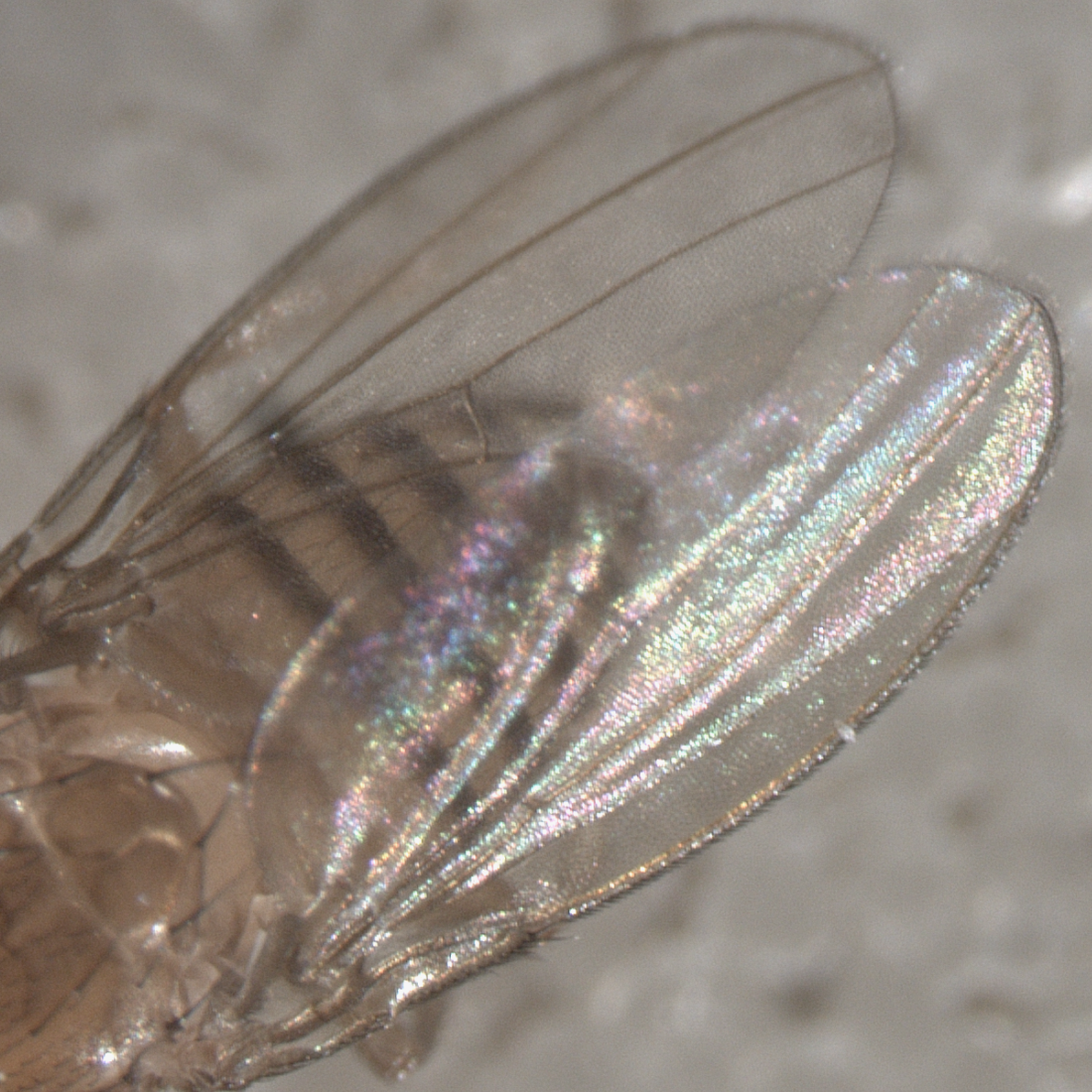

Supplement: Supplementary file 15 — Source data Fig. 8 [file 44318_2025_384_MOESM15_ESM.zip › Figure 8/fig8_h.tif]

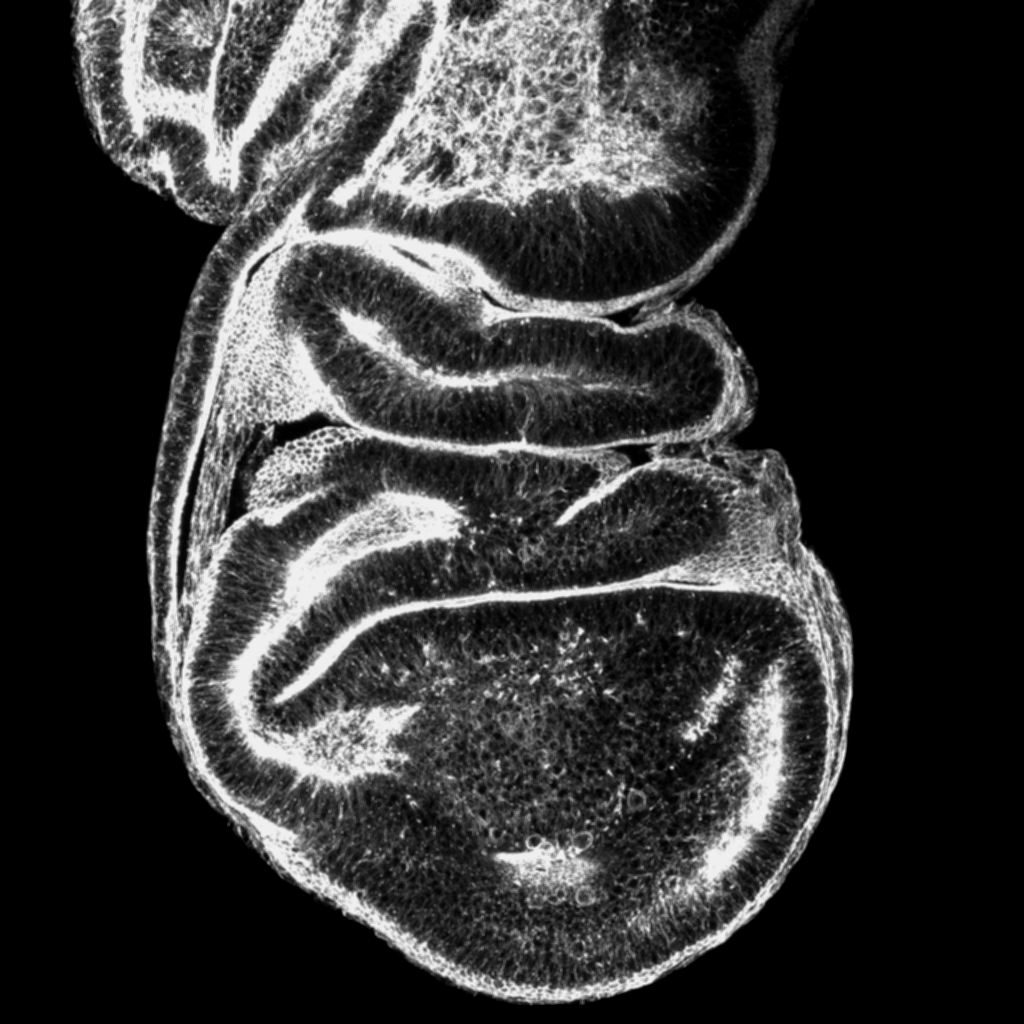

Supplement: Supplementary file 15 — Source data Fig. 8 [file 44318_2025_384_MOESM15_ESM.zip › Figure 8/fig8_e.tif]

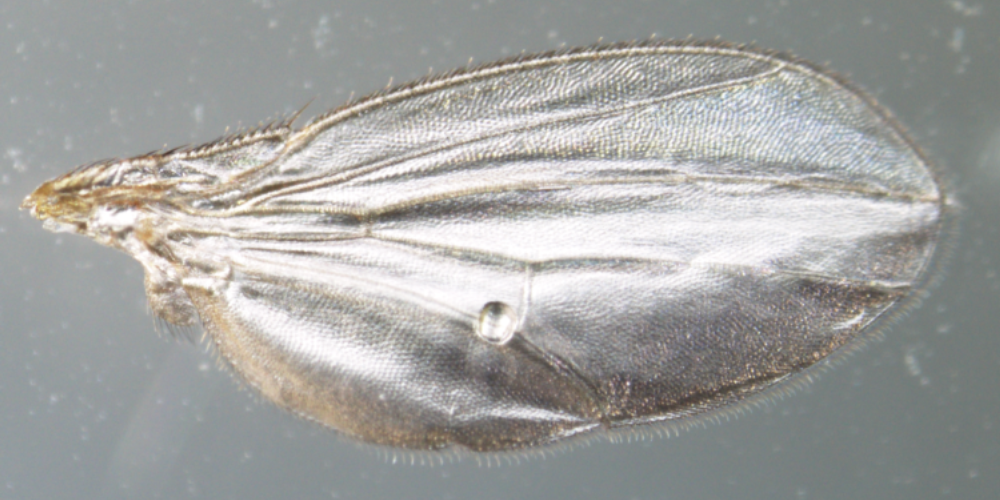

Supplement: Supplementary file 15 — Source data Fig. 8 [file 44318_2025_384_MOESM15_ESM.zip › Figure 8/fig8_d.tif]

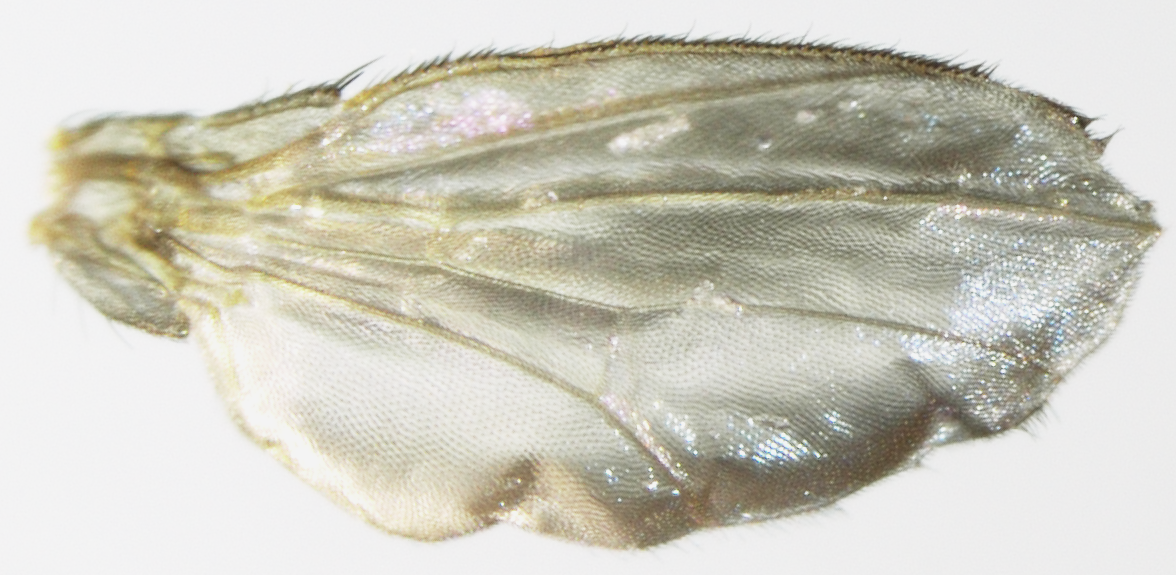

Supplement: Supplementary file 15 — Source data Fig. 8 [file 44318_2025_384_MOESM15_ESM.zip › Figure 8/fig8_f.tif]

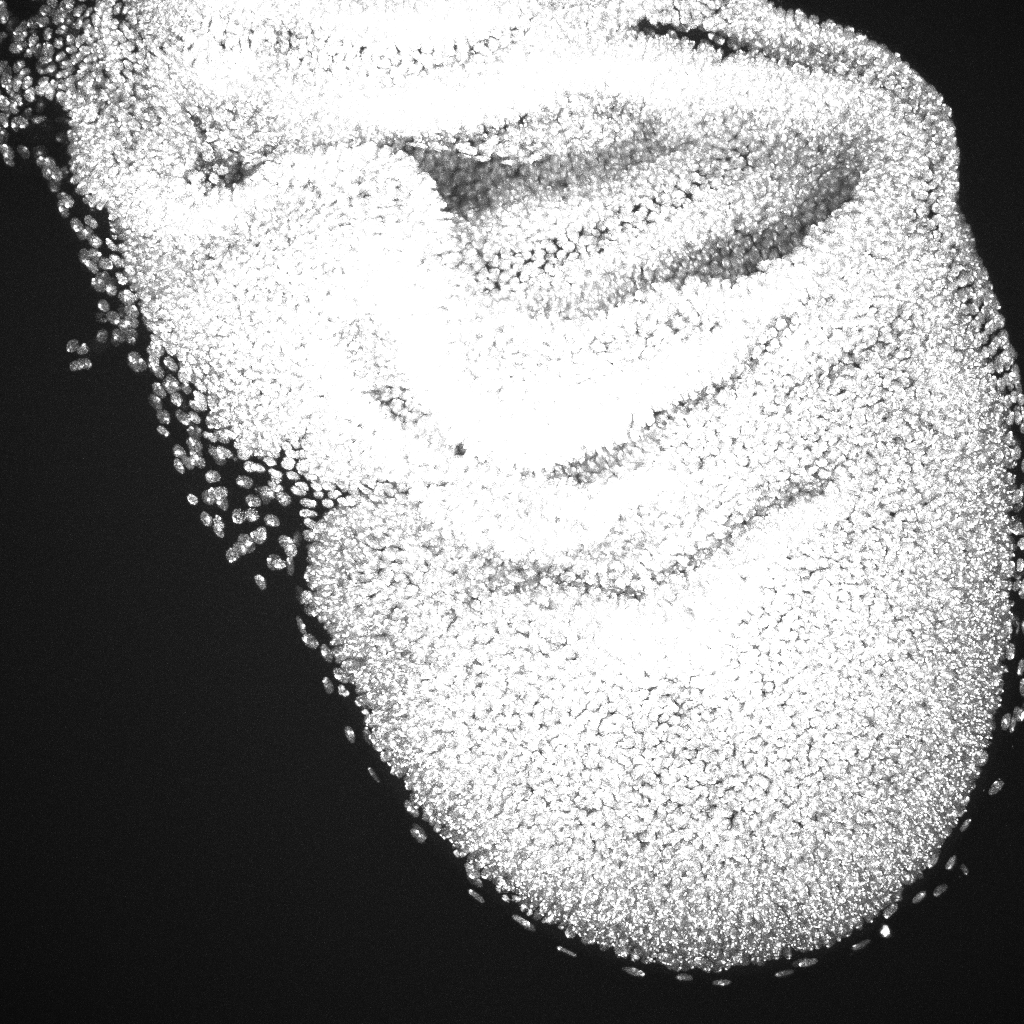

Supplement: Supplementary file 15 — Source data Fig. 8 [file 44318_2025_384_MOESM15_ESM.zip › Figure 8/fig8_g.tif]

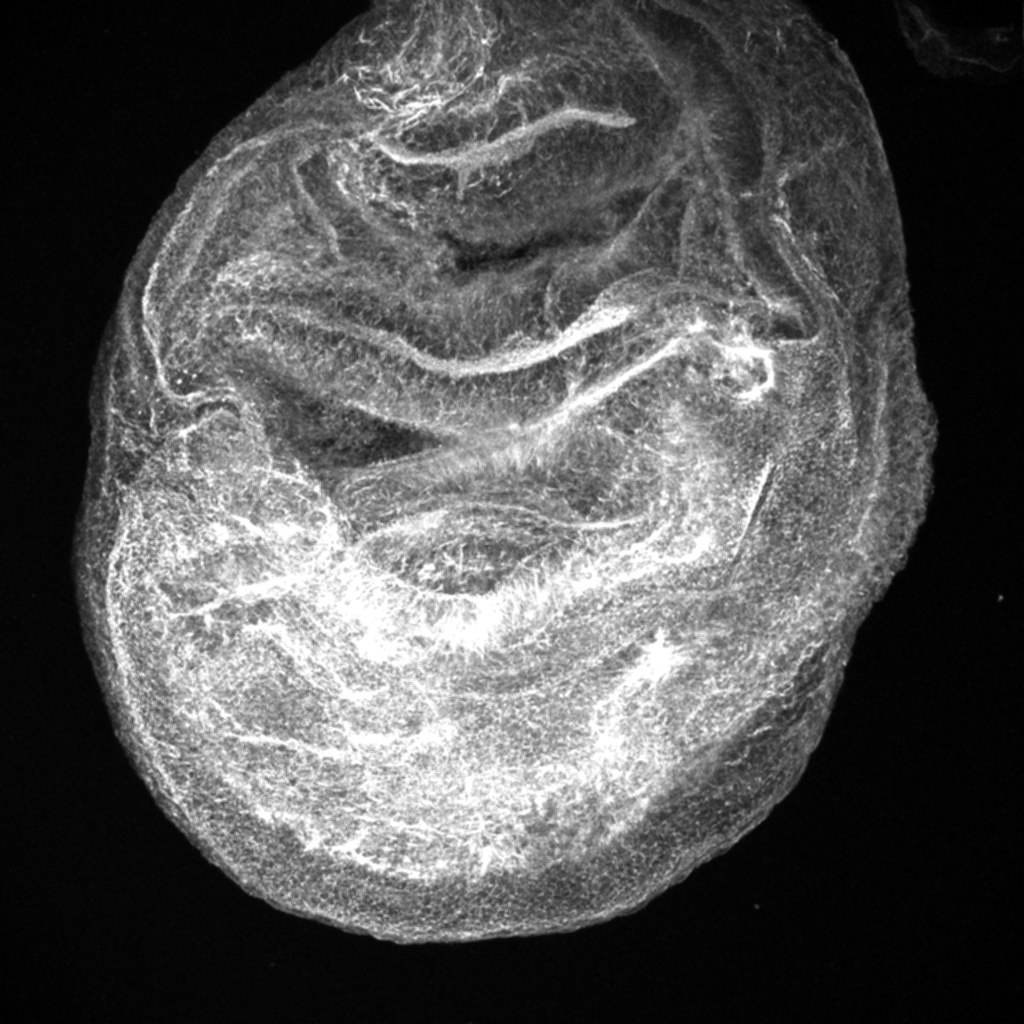

Supplement: Supplementary file 15 — Source data Fig. 8 [file 44318_2025_384_MOESM15_ESM.zip › Figure 8/fig8_c.tif]

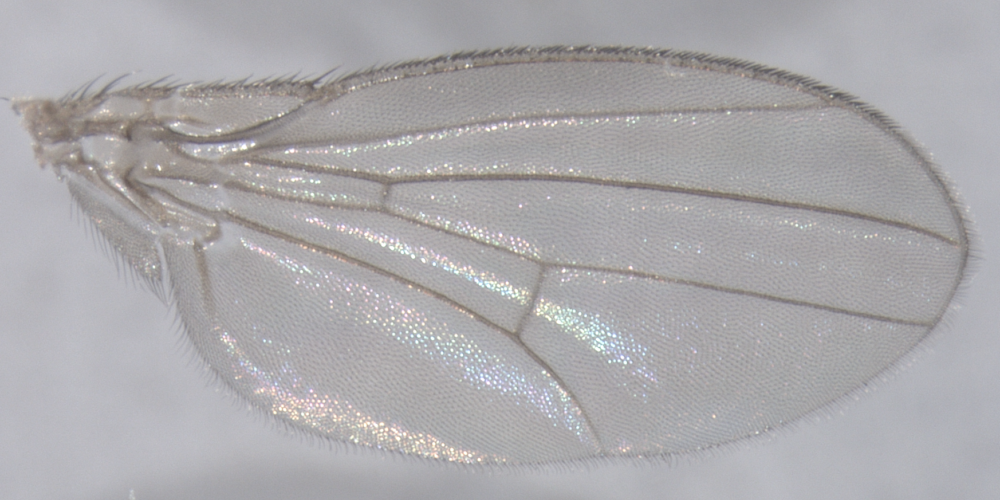

Supplement: Supplementary file 15 — Source data Fig. 8 [file 44318_2025_384_MOESM15_ESM.zip › Figure 8/fig8_B.tif]

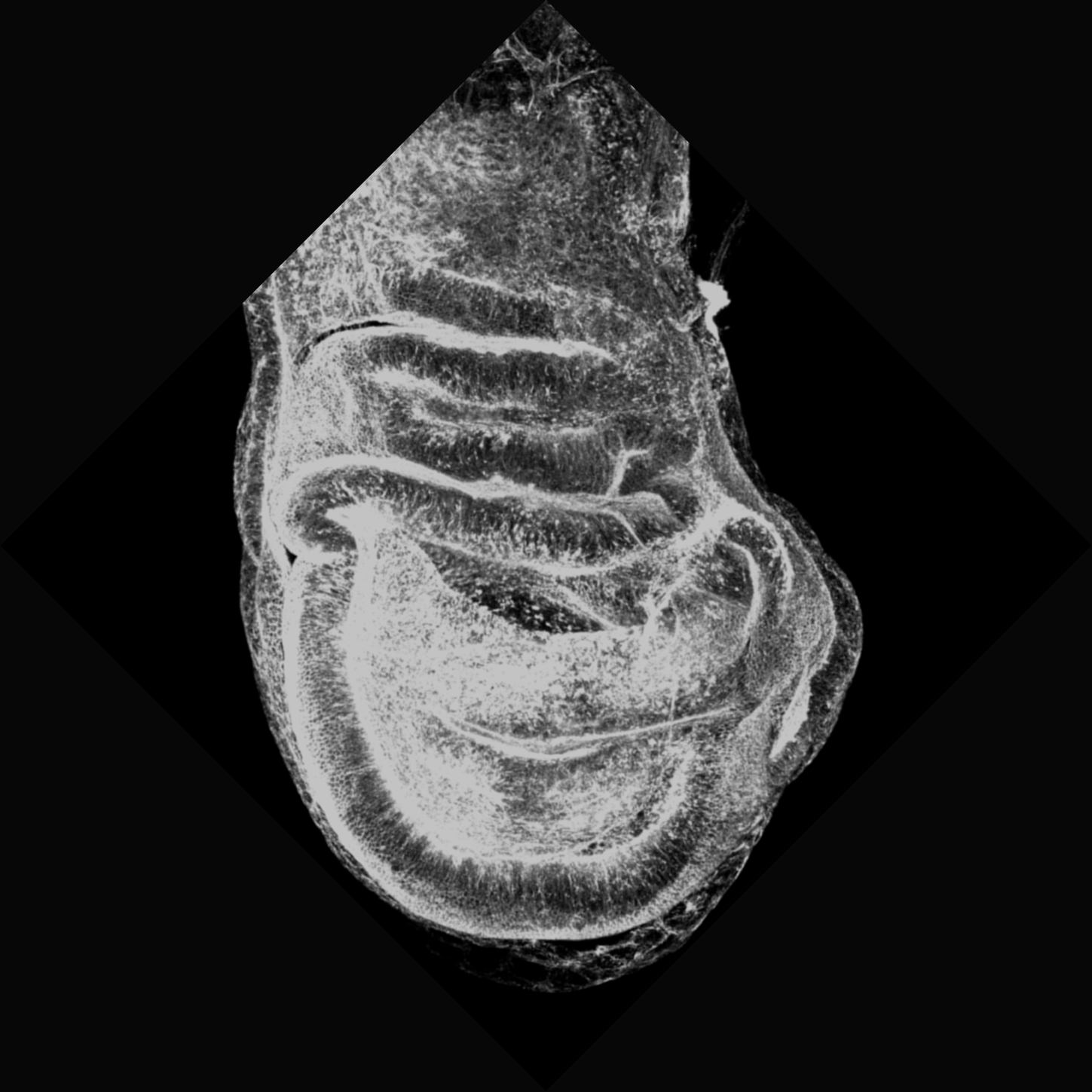

Supplement: Supplementary file 15 — Source data Fig. 8 [file 44318_2025_384_MOESM15_ESM.zip › Figure 8/fig8_a.tif]
