# Supplementary material for: Local weakening of cell-extracellular matrix adhesion triggers basal epithelial tissue folding
Source: EMBO J. 2025 Feb 17;44(7):2002–24. doi: 10.1038/s44318-025-00384-6 (PMC11961693; doi:10.1038/s44318-025-00384-6)
Supplement: Supplementary file 17 — Expanded View Figures [file 44318_2025_384_MOESM17_ESM.pdf]

Expanded View Figures

Figure EV1. F-actin and Myosin organization changes throughout development in wing margin cells.

(**A–D**) Confocal views of third-instar wing discs stained with anti-mys-GFP (green in **A–A′**, **B–B′**, **C–C′**, **D–D′** and white in **A″**, **B″**, **C″**, **D″**) and Rhodamine Phalloidin to detect F-actin (magenta in **A–A′**, **B–B′**, **C–C′**, **D–D′** and white in **A‴**, **B‴**, **C‴**, **D‴**). (**A**, **B**, **C**, **D**) Maximal projections of 80 h AED (**A**, **C**) and 96 h AED (**B**, **D**) wing discs. (**A′–A‴**, **B′–B‴**, **C′–C‴**, **D′–D‴**) High resolution images of YZ sections taken at the region in the dotted square in (**A**, **B**, **C**, **D**), respectively. (**E**) Quantification of *mys*-GFP, *sqh*-GFP and F-actin levels in mid L3 wing discs at the regions framed in (**A′**, **B′**, **C′**, **D′**). Multiple Mann–Whitney U test from left to right: ***$p = 0.0002$, ***$p = 0.0002$, ***$p = 0.0016$, ***$p = 0.0049$, ***$p = 0.0003$, ***$p = 0.0007$. Error bars represent the mean ± SEM. Scale bar in all panels, 30 μm. At least 15 wing discs were assessed over three independent experiments. Source data are available online for this figure.

© The Author(s)

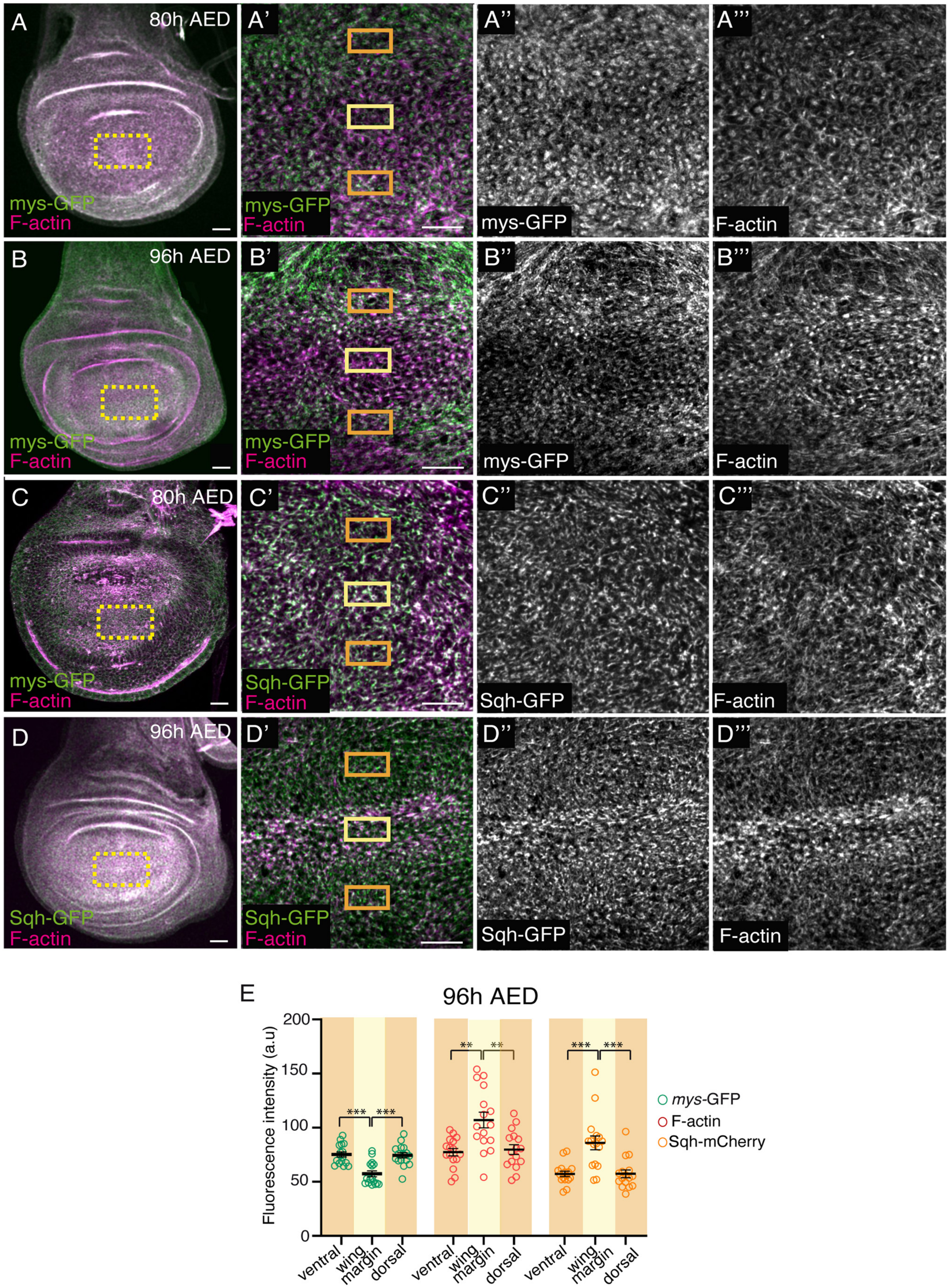
A
80h AED
mys-GFP
F-actin
A'
A''
A'''
B
96h AED
B'
B''
B'''
C
C'
C''
C'''
Sqh-GFP
D
D'
D''
D'''
E
96h AED
Fluorescence intensity (a.u)
200
150
100
50
0
***
**
ventral
wing margin
dorsal
mys-GFP
F-actin
Sqh-mCherry

 © The Author(s)

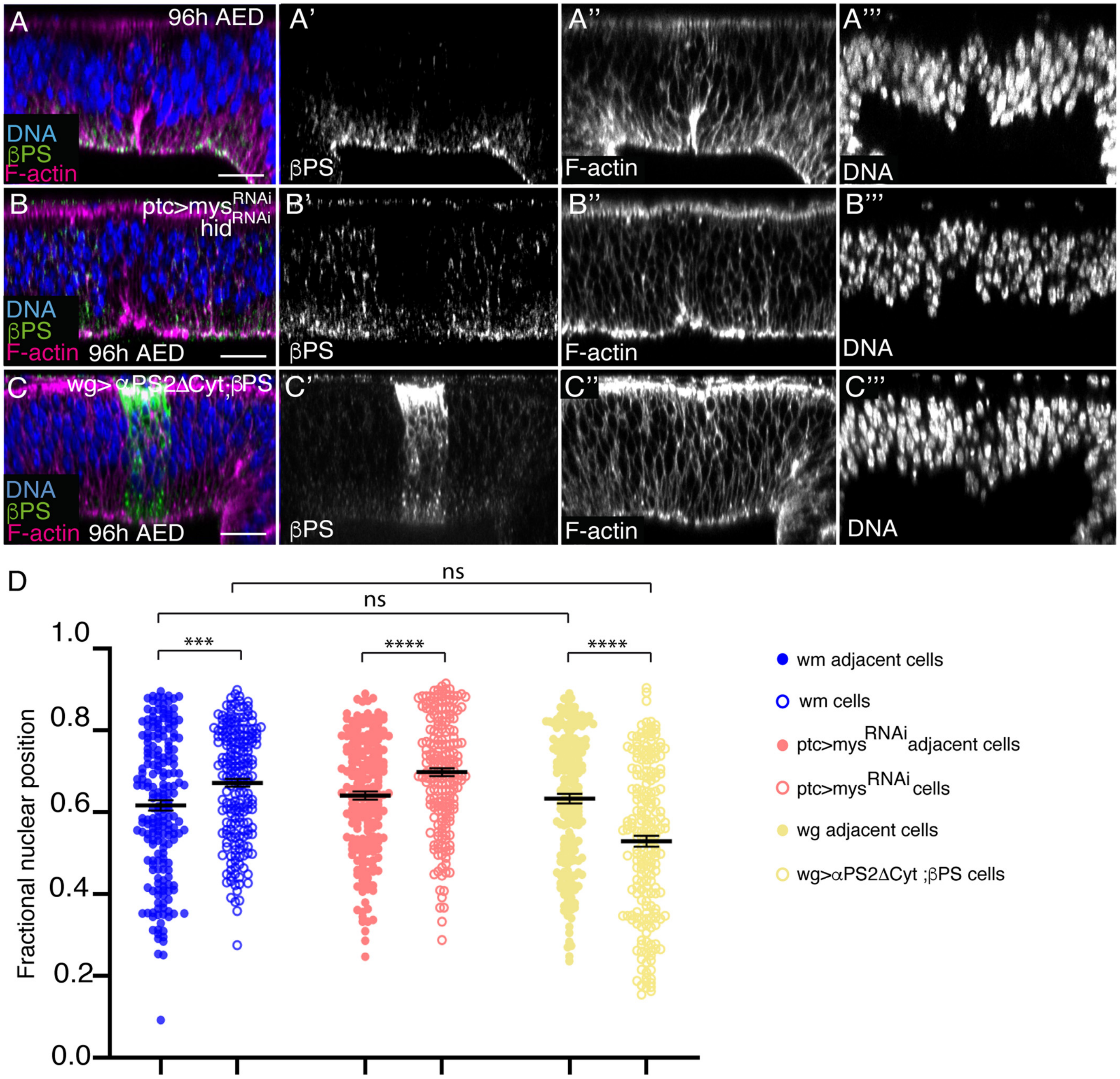

Figure EV2. Integrin levels condition nuclear positioning.

Confocal YZ (**A–A**″, **C–C**″) and XZ (**B–B**″) cross-sections of third-instar wing discs stained with anti-βPS (green in **A**, **B**, **C** and white in **A**′, **B**′, **C**′), Rhodamine Phalloidin to detect F-actin (magenta in **A**, **B**, **C** and white in **A**″, **B**″, **C**″) and the nuclear marker Hoechst (DNA, blue in **A**, **B**, **C** and white in **A**‴, **B**‴, **C**‴). (**A**) Control wing disc. (**B**) Wing disc co-expressing RNAis against *mys* and *hid* under the control of *ptcGal4*, *ptc>mys*^{RNAi}; *hid*^{RNAi}. (**C**) Wing disc co-expressing an active form of the αPS2 subunit and the βPS subunit under the control of *wgGal4*, *wg>αPS2ΔCyt; βPS*. (**D**) Quantification of the fractional nuclear position in wing margin and adjacent cells in control and experimental wing discs. Multiple Mann–Whitney U test from left to right: ***$p = 0.000455$, ****$p = 1.62768E-05$, ****$p = 7.05405E-09$, ns not significant. Error bars represent the mean ± SEM. Scale bar in all panels, 30 µm. 200 nuclei were analyzed in at least 15 wing discs over three independent experiments. Source data are available online for this figure.

© The Author(s)

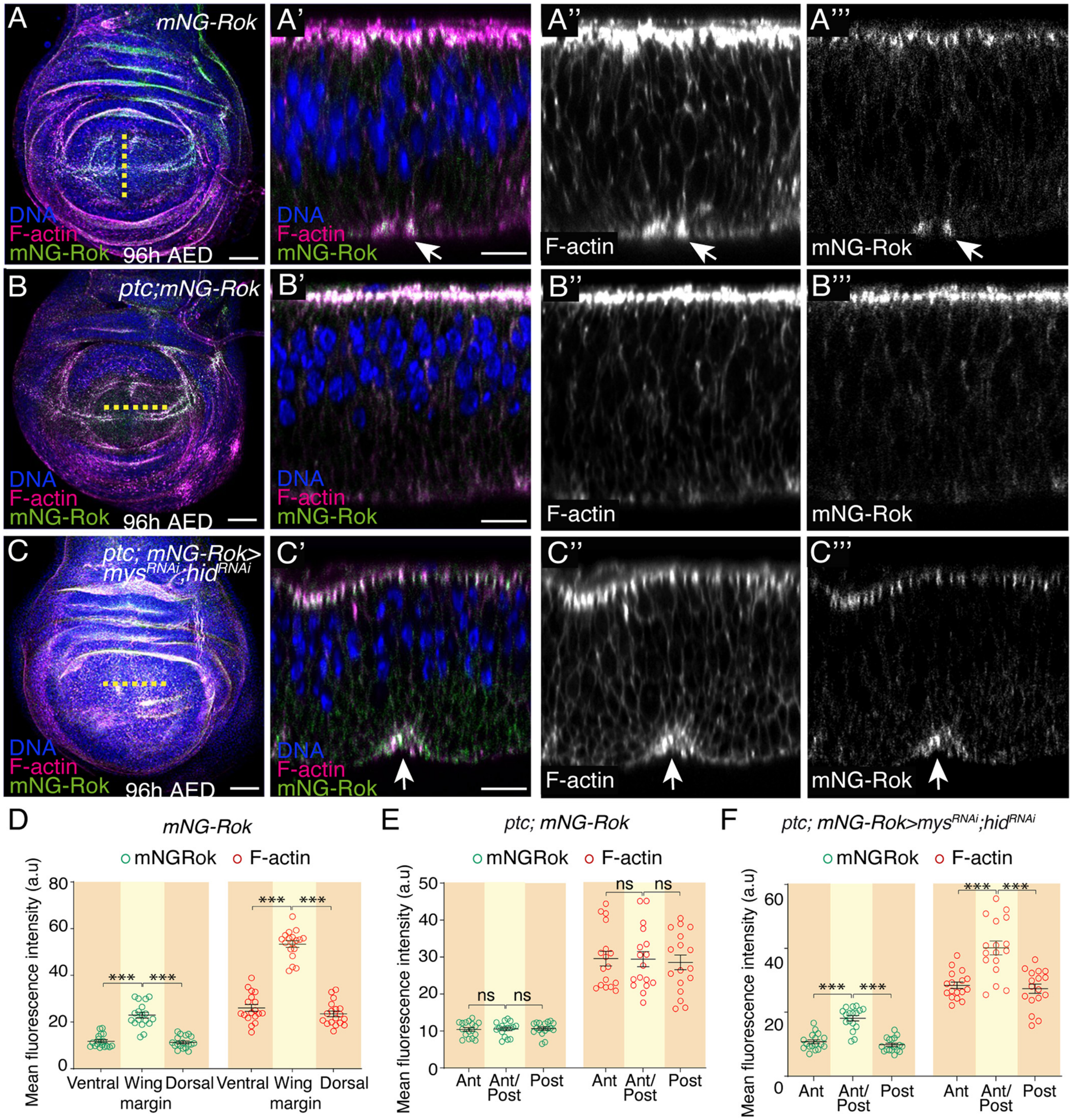

Figure EV3. Rok is found basally in wing cells with low integrin levels.

(**A–C**) Confocal views of third-instar wing discs of the indicated genotypes, stained with Rhodamine Phalloidin to detect F-actin (magenta **A**, **A′**, **B**, **B′**, **C**, **C′** and white in **A″**, **B″**, **C″**) and the nuclear marker Hoechst DNA (blue in **A**, **A′**, **B**, **B′**, **C**, **C′**). *mNGROK* signal is shown in green in **A**, **A′**, **B**, **B′**, **C**, **C′** and in white in **A‴**, **B‴**, **C‴** (**A–C**). Maximal projections of a third-instar wing imaginal discs of the indicated genotypes. Confocal YZ (**A′–A‴**) and XZ (**B′–B‴**, **C′–C‴**) cross-sections along the yellow dotted lines shown in (**A–C**). (**D–F**) Quantification of *mNGROK* levels in controls and experimental wing discs along the white dotted lines in (**A**), (**B**) and (**C**). Multiple Mann–Whitney U test from left to right: (**D**) ***$p = 0.002$, ***$p = 0.001$, ***$p = 0.0006$, ***$p = 0.00011$, (**E**) ns not significant, (**F**) ***$p = 0.0013$, ***$p = 0.00011$, ***$p = 0.00074$, ***$p = 0.00047$. Error bars represent the mean ± SEM. Scale bar in all panels, 30 μm. At least 15 wing discs were assessed over three independent experiments. Source data are available online for this figure.

 © The Author(s)

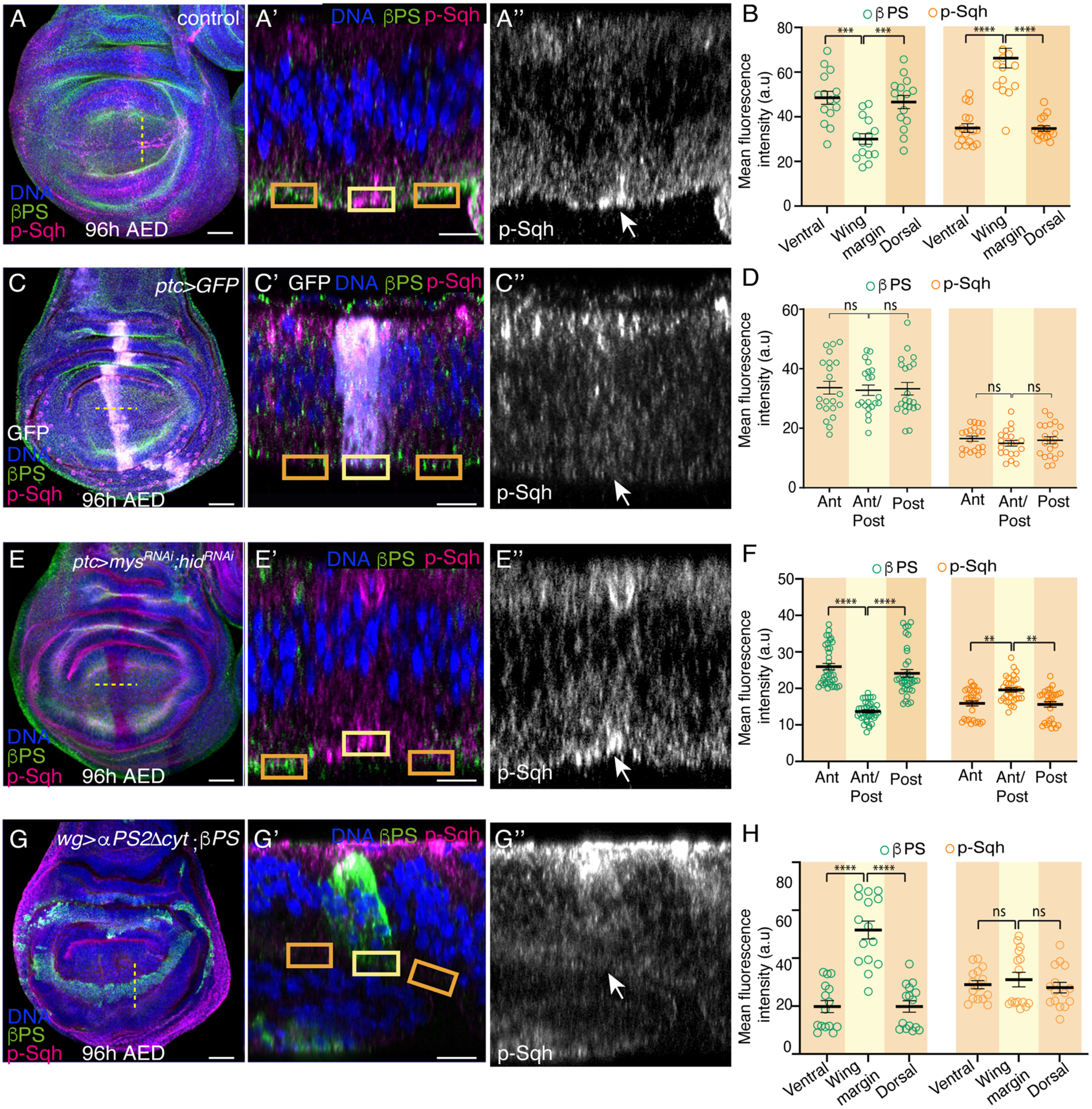

Figure EV4. Integrins regulate pSqh levels in wing margin cells.

(**A–A″, C–C″, E–E″, G–G″**) Confocal views of third-instar wing discs of the designated genotypes stained with anti-βPS (green), anti-pSqh (magenta in **A**, **A′**, **C**, **C′**, **E**, **E′**, **G**, **G′** and white in **A″**, **C″**, **E″**, **G″**), and the nuclear marker Hoechst DNA (blue in **A**, **A′**, **C**, **C′**, **E**, **E′**, **G**, **G′**). (**A**, **C**, **E**, **G**) Maximal projections of a third-instar wing imaginal discs of the indicated genotypes. Confocal YZ (**A′**, **A″**, **G′**, **G″**) and XZ (**C′**, **C″**, **E′**, **E″**) cross-sections along the yellow dotted lines shown in (**A**, **C**, **E**, **G**). (**B**, **D**, **F**, **H**) Quantification of βPS and pSqh levels in control and experimental wing discs in the regions framed in (**A′**, **C′**, **E′**, **G′**) (orange and yellow boxes). Multiple Mann–Whitney U test from left to right: (**B**) ***$p = 0.00013$, ***$p = 0.0029$, ****$p = 1.1e{-}5$, ****$p = 1.1e{-}5$, (**D**) ns not significant, (**F**) ****$p = 4.4e{-}14$, ****$p = 6.1e{-}11$, **$p = 0.0024$, **$p = 0.0018$, (**H**) ****$p = 4.8e{-}14$, ****$p = 6.1e{-}11$, ns not significant. Error bars represent the mean ± SEM. Scale bar in all panels, 30 μm. At least 15 wing discs were assessed over three independent experiments. Source data are available online for this figure.

© The Author(s)
